# Head-to-Head Comparison between Collagen Proportionate Area and Acoustic Radiation Force Impulse Elastography in Liver Fibrosis Quantification in Chronic Hepatitis C

**DOI:** 10.1371/journal.pone.0140554

**Published:** 2015-10-13

**Authors:** Sheng-Hung Chen, Cheng-Yuan Peng, Hsueh-Chou Lai, I-Ping Chang, Chiung-Ju Lee, Wen-Pang Su, Chia-Hsin Lin, Jung-Ta Kao, Po-Heng Chuang

**Affiliations:** 1 Graduate Institute of Clinical Medical Science, School of Medicine, China Medical University, Taichung, Taiwan; 2 School of Medicine, China Medical University, Taichung, Taiwan; 3 Division of Hepatogastroenterology, Department of Internal Medicine, China Medical University Hospital, Taichung, Taiwan; 4 College of Chinese Medicine, China Medical University, Taichung, Taiwan; 5 Department of Pathology, China Medical University Hospital, Taichung, Taiwan; National Taiwan University Hospital, TAIWAN

## Abstract

**Background:**

The aim of this study was to compare the diagnostic performances of the collagen proportionate area (CPA) and liver stiffness measurement (LSM) for liver fibrosis quantification in chronic hepatitis C (CHC).

**Methods:**

A total of 137 eligible consecutive Taiwanese patients (74 women and 63 men; age 21–80 years; median age 54 years), with CHC underwent LSM by using acoustic radiation force impulse (ARFI) elastography and an immediate percutaneous liver biopsy for METAVIR scoring. Liver tissue sections were stained using picrosirius red. Areas of the stained collagen and the tissue parenchyma were calculated in pixels. The ratio between the two areas was expressed as a CPA percentage. The result of LSM was presented as shear wave velocity (SWV).

**Results:**

METAVIR fibrosis (F) stages were dichotomized using the CPA (%) and SWV (m/s), and the optimal cut-off values were 7.47 and 1.59 for F1 versus F2–4; 12.56 and 1.73 for F1, 2 versus F3, 4; 15.32 and 1.96 for F1–3 versus F4. To dichotomize F1 versus F2–4, the areas under receiver operating characteristic curves for the CPA was 0.9349 (95% confidence interval: 0.8943–0.9755) and for SWV was 0.8434 (0.7762–0.9105) (CPA versus SWV, *P* = 0.0063). For F1, 2 versus F3, 4, the CPA was 0.9436 (0.9091–0.9781); SWV was 0.8997 (0.8444–0.9551) (*P* = 0.1587). For F1–3 versus F4, the CPA was 0.8647 (0.7944–0.9349); SWV was 0.9036 (0.8499–0.9573) (*P* = 0.2585). The CPA could be predicted in a linear regression formula by using SWV and platelet count (R^2^ = 0.524).

**Conclusions:**

The CPA and ARFI elastography are promising tools for liver fibrosis evaluation. The CPA was superior to ARFI elastography in the diagnosis of significant fibrosis (≥ F2). The CPA may be independent of severe necroinflammation, which may augment liver stiffness.

## Introduction

Chronic hepatitis C (CHC) is a major health care burden and a leading cause of end-stage liver disease and hepatocellular carcinoma (HCC) worldwide [1].

The immune response is insufficient to eradicate hepatitis C virus (HCV) when the chronic infection is established and chronic liver necroinflammation and progressive fibrogenesis are triggered. The maladaptive healing process interferes with liver function by increasing extracellular matrix depositions, compromising substance diffusion, worsening portal resistance, and increasing the risk of tumorigenesis. The severity of fibrogenesis is correlated to adverse treatment effects and inversely correlated to treatment response.

In the last decade, new molecular insights into fibrogenesis and potential therapeutic targets for fibrosis regression necessitate noninvasive modalities for measuring progression or reversal in fibrosis [2].

Liver stiffness measurement (LSM) by using acoustic radiation force impulse (ARFI) elastography is a noninvasive solution for liver fibrosis evaluation in preliminary observations and is predominantly implemented in CHC [3]. Despite the promising reproducibility and validity reported, caution and concern still remain fundamental regarding the variations in measurement results. These variations are attributable to ethnicity, systems, measurement techniques, steatosis [4], and the mathematically unpredictable augmentation of liver stiffness (LS) because of hepatic necroinflammation, jaundice, and cardiac congestion [3].

The diagnostic validity of ultrasound-based LSM increases with fibrosis stage. Necroinflammation can severely compromise validity when dichotomizing liver fibrosis stages to diagnose significant fibrosis (METAVIR fibrosis stage ≥ 2) [5, 6]. Previous studies have been mostly consistent regarding the positive effects of hepatic necroinflammation on LS; however, results varied [7].

In addition to an invasive liver pathology staged and graded using the METAVIR scoring system, determining the collagen proportionate area (CPA) by using digital image analysis has rarely been applied in clinical practice because of its invasiveness. Nonetheless, the CPA is highly correlated to conventional fibrosis staging [8, 9, 10], the hepatic vein pressure gradient [10, 11, 12], LS [9], cirrhosis stage [12, 13], and prognosis [10, 14].

Moreover, when explaining and reexamining the effects of necroinflammation on LS, the CPA may be superior to METAVIR F because of its quantitative consistency despite the relatively subjective morphometric thresholding processes involved in digital image analysis.

Few studies have compared the diagnostic performances of the CPA with those of the LSM by using transient or point shear wave elastography [9, 15, 16]. No study has compared head-to-head the diagnostic performances of the CPA with those of the LSM using ARFI. No study has provided a formula by using noninvasive LSM to predict CPA, which can be applied as a practical index for liver fibrosis quantification in clinical and research settings.

Therefore, our aims were to compare head-to-head the liver fibrosis quantification by using the CPA and LS, to reexamine the necroinflammatory effects on LS after adjusting for CPA rather than conventional fibrosis stage, and to predict CPA by using LS.

## Materials and Methods

### Ethics statement

The study protocol conforms to the ethical guidelines of the 1975 Declaration of Helsinki. The protocol was approved by the Research Ethics Committee of China Medical University Hospital (CMUH103-REC1-008). Written informed consent was obtained from all patients included in the study.

### Patients

Consecutive Taiwanese patients diagnosed with CHC from January 2013 to January 2015 were screened and enrolled in a prospective cohort for the analysis of antiviral treatment responses. CHC infections were defined as serum positive for the anti-HCV antibody (Abbott Laboratories, Abbott Park, IL, USA) for more than 6 months with detectable serum HCV RNA (detection limit: 15 IU/mL; COBAS Ampliprep/COBAS TaqMan HCV test, Roche Diagnostics, Branchburg, NJ, USA). Patient exclusion criteria were as follows: age <20 years, hepatitis B virus coinfection, human immunodeficiency virus coinfection, decompensated liver cirrhosis, HCC, alcoholic liver disease, primary biliary cirrhosis, primary sclerosing cholangitis, Wilson disease, autoimmune hepatitis, hemochromatosis, extrahepatic cholestasis, myeloproliferative disorders, thalassemia, cardiac congestion, blood product transfusion in the preceding 30 days, pregnancy, and serum creatinine higher than 221 μmol/L (2.5 mg/dL).

### Blood tests

Blood biochemistry (Beckman Coulter, CA, USA) and complete blood count analyses (Sysmex HST-series, Kanogawa, Japan) were performed in the same laboratory of the medical center. The HCV RNA was quantified at baseline (COBAS Ampliprep/COBAS TaqMan HCV test, Roche Diagnostics, Branchburg, NJ, USA). HCV genotyping was performed by direct sequence analysis of a 244-bp fragment of the 5’ untranslated region generated with polymerase chain reactions according to the method described previously [17]. Aspartate transaminase (AST)-to-platelet ratio index (APRI) = (AST / upper limit of normal, 34 IU/L) / platelet count (10^9^/L) × 100.

### LSM using ARFI

Participants underwent percutaneous liver biopsy within 1 hour of blood tests and stiffness measurements preceded by 3 hours of fasting [18].

ARFI technology was integrated into a conventional ultrasound system (Acuson S2000 with a Siemens 4C1 curved array, 4.00 MHz for B-mode, 2.67 MHz for push pulses, and 3.08 MHz for detection pulses; Siemens Medical Solutions, Mountain View, CA, USA). The detection pulses measured the tissue stiffness as shear wave velocity (SWV) in meters per second.

All ARFI stiffness measurements were performed by the same hepatologist experienced in digestive system ultrasonography and blinded to patient data. Reliable cases were defined as those with an interquartile range (IQR) lower than 30% of the median of 10 successful LSMs, and a successful LSM rate higher than 60%. Other cases were considered unreliable and excluded.

### METAVIR scoring

Senior hepatologists performed percutaneous right lobe liver biopsy. The biopsy specimens were stained using Masson trichrome, hematoxylin and eosin, and reticulin stains and interpreted by an experienced pathologist blinded to the LSM results and patient data. Biopsy specimens at least 15 mm in length containing at least five portal tracts were defined as adequate [19]. Liver fibrosis (F) was staged as no fibrosis (F0), portal fibrosis without septa (F1), portal fibrosis with a few septa (F2), numerous septa without cirrhosis (F3), and cirrhosis (F4). Necroinflammatory activity (A) was graded as none (A0), mild (A1), moderate (A2), and severe (A3) [5]. Steatosis was scored using a scoring system as no steatosis (S0); mild (S1), 1%–5% hepatocytes containing visible macrovesicular steatosis; moderate (S2), 6%–32%; marked (S3), 33%–66%; severe (S4), 67%–100% [20].

### Collagen proportionate area

The CPA was measured as described previously [11, 21, 22]. Liver tissue sections were stained using picrosirius red 0.1% (w/v), and digital images were captured using a digital camera (Canon EOS 650D, Tokyo, Japan) connected to a desktop computer. The images were edited using the Adobe Photoshop CS6 software platform (Adobe Systems, Inc., San Jose, CA, USA) to exclude collagenous structures irrelevant to hepatitis, including collagen in the walls of the portal tracts and other blood vessels. Interactive thresholding was completed through consensuses between the hepatologists and the pathologist. The stained collagen and the tissue areas were edited and calculated in pixels by using Image-Pro Plus Version 7.0 (Media Cybernetics, Inc., Rockville, MD, USA). The ratio between the two areas was expressed as a CPA percentage.

### Statistical analysis

Between-group and overall differences were estimated using the Mann-Whitney U test and Kruskal-Wallis test for continuous variables and chi-square test or Fisher's exact test for proportions, respectively. Spearman rank correlation was used to evaluate the significance of correlations between two variables.

Receiver operating characteristic (ROC) analysis was used to optimize the cut-off values and evaluate diagnostic performances by using areas under the ROC curves (AUROC). The AUROCs between the CPA and SWV were compared [23].

The variables of age, sex, body mass index, CPA, SWV, hepatic METAVIR F stages, A grades, steatosis grades, HCV genotype, HCV RNA viral load, white blood cell count, platelet count, international normalized ratio of prothrombin time (INR), hemoglobin level, serum alanine transaminase (ALT), albumin, bilirubin, creatinine, and sodium levels were designated as covariates in the univariate and multiple regression analyses.

Variables with a *P* value of less than 0.25 after univariate linear regression were included in stepwise multiple linear regression modeling. Finally, multiple linear regressions were used to identify significant independent explanatory factors for the CPA and SWV, respectively. Data were analyzed using SAS Version 9.3 (SAS Institute, Inc., Cary, NC, USA) and SPSS Version 17.0 for Microsoft Windows (SPSS, Chicago, IL, USA). A two-sided *P* value of < 0.05 indicated statistical significance.

## Results

### Participants

A total of 156 patients were screened. Patients with hepatitis B virus coinfection (n = 7), HCC (n = 2), and alcoholic liver disease (n = 5) were excluded. Five cases were excluded for unreliable LSMs.

Thus, a cohort of 137 patients (74 (54%) women and 63 (46%) men aged 21–80 years (median 54 years)) was analyzed (Table 1). A total of 71 (51.8%) and 66 (48.2%) patients were infected with HCV of genotypes non-1 and 1, respectively.

**Table 1:** Patient characteristics.

	METAVIR		
Variable	F1–3	F4	*P* value
Age (years)	53(45.5–60)	59.5(53–66)	0.002
Sex			
Female/male	56(49.6)/57(50.4)	18(75.0)/6(25.0)	0.023
Body mass index (kg/m^2^)	24.1(22.4–26.2)	24.5(22.2–25.7)	0.917
HCV genotype			
1/2/3/6	53(46.9)/52(46.0)/1(0.9)/7(6.2)	18(75.0)/5(20.8)/0/1(4.2)	0.094
HCV RNA (IU/mL)	3765000(431750–9705000)	3075000(828750–8480000)	0.646
METAVIR Fibrosis stage			
0/1/2/3/4	0/34(30.1)/45(39.8)/34(30.1)	24	
METAVIR Activity grade			
0/1/2/3	17(15.0)/61(54.0)/32(28.3)/3(2.7)	0/13(54.2)/11(45.8)/0	0.103
Steatosis grade			
0/1/2/3/4	18(15.9)/38(33.6)/55(48.7)/2(1.8)/0	0/7(29.2)/17(70.8)/0/0	0.101
Alanine transaminase (IU/L)	87(54–146)	100(74–142)	0.292
Albumin (g/dL)	4.3(4.1–4.6)	3.6(4.0–4.2)	<0.001
Bilirubin (μmol/L)	17.1(13.7–19.7)	20.5(15.4–26.9)	0.022
Creatinine (μmol/L)	67.2(57.0–79.6)	58.8(52.6–83.8)	0.276
Hemoglobin (g/dL)	14.3(13.4–15.3)	13.3(12.7–14.5)	0.001
International normalized ratio^a^	1.05(0.99–1.11)	1.15(1.07–1.22)	<0.001
Platelet (10^9^/L)	167(118–208)	107(76–121)	<0.001
Sodium (mEq/L)	138(137–140)	139(137–140)	0.241
White blood cell (/mm^3^)	5130(4310–5905)	4745(4130–5738)	0.388
CPA^b^ distribution^c^			
F^d^1	3.99 (0.58–10.32)		
F2	9.03 (1.25–25.82)		
F3	24.48 (7.68–56.64)		
F4	29.42 (8.37–64.21)		
SWV^e^ distribution^c^			
F1	1.21 (1.00–2.43)		
F2	1.54 (0.98–3.58)		
F3	2.37 (0.83–4.05)		
F4	3.04 (1.98–4.56)		

^a^International normalized ratio of prothrombin time;

^b^collagen proportionate area (%); data are presented as medians (interquartile range or range^c^) or No (%);

^d^METAVIR Fibrosis stage;

^e^shear wave velocity (m/s).

### Liver histology

**Fig 1:**
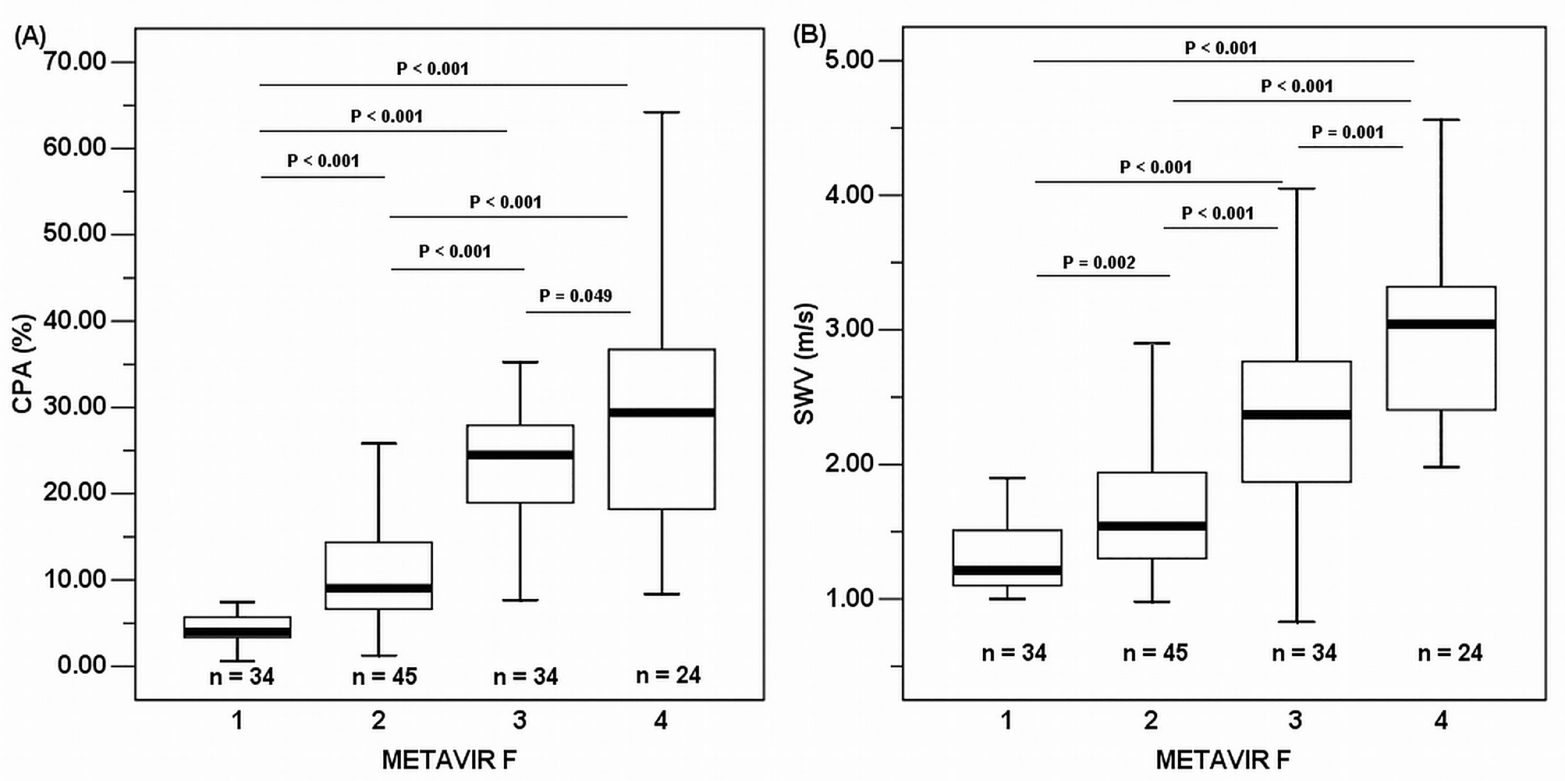
Box plots of the CPA^a^ (A) and SWV^b^ (B). ^a^collagen proportionate area; ^b^shear wave velocity.

According to the METAVIR F scoring system, 34, 45, 34, and 24 participants were staged as F1, F2, F3, and F4, respectively. According to the METAVIR A system 17, 74, 43, and 3 participants were graded as A0, A1, A2, and A3, respectively. The Spearman rank correlation coefficient between the ALT levels and METAVIR A was 0.655 (*P* < 0.001). According to steatosis grading, 18, 45, 72, and 2 participants were graded as S0, S1, S2, and S3, respectively (Table 1 and Fig 1).

### APRI for dichotomizing fibrosis stages

**Fig 2:**
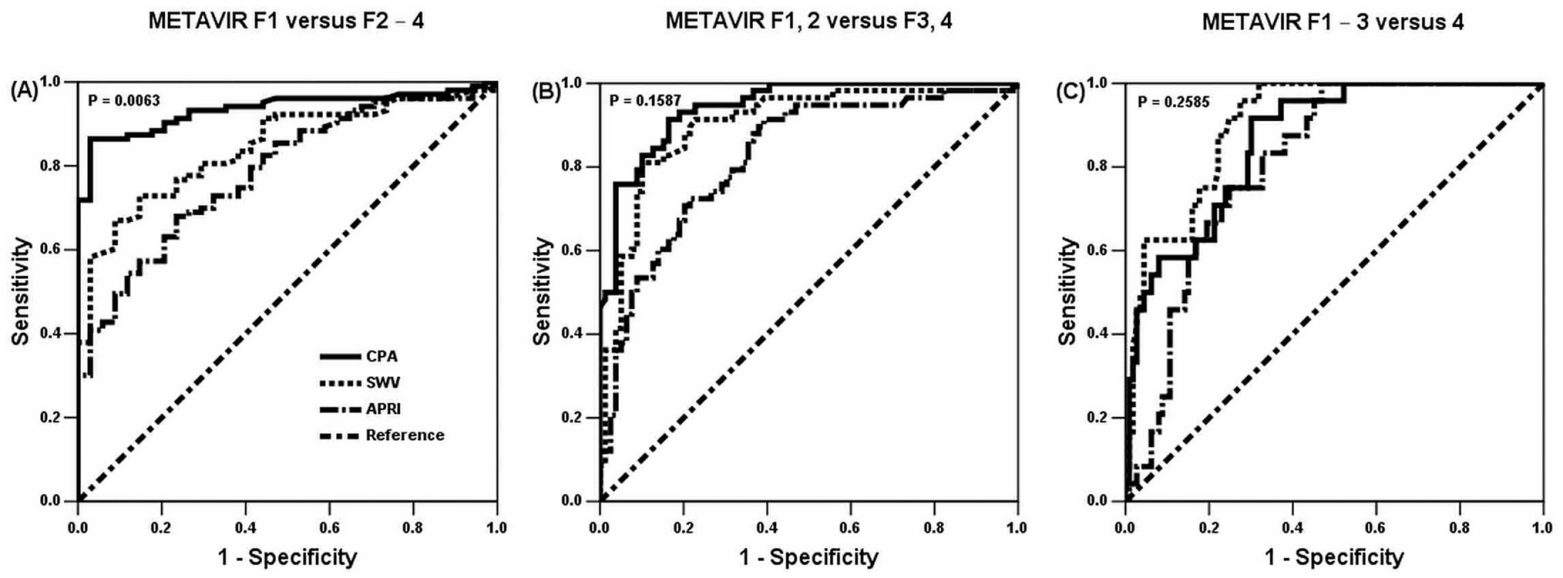
ROC^a^ curves dichotomizing liver fibrosis stages. To dichotomize F1 versus F2–4 (A), the AUROC^b^ for the CPA^c^ was 0.9349 (95% confidence interval: 0.8943–0.9755) and SWV^d^ was 0.8434 (0.7762–0.9105) (CPA versus SWV, *P* = 0.0063). For F1, 2 versus F3, 4 (B), the CPA was 0.9436 (0.9091–0.9781); SWV was 0.8997 (0.8444–0.9551) (*P* = 0.1587). For F1–3 versus F4 (C), the CPA was 0.8647 (0.7944–0.9349); SWV was 0.9036 (0.8499–0.9573) (*P* = 0.2585). APRI^e^ was incorporated too. ^a^receiver operating characteristic; ^b^area under ROC curves; ^c^collagen proportionate area; ^d^shear wave velocity; ^e^aspartate transaminase-to-platelet ratio index.

To dichotomize F1 versus F2–4, the AUROC for the APRI was 0.7846 (95% confidence interval (CI): 0.7033–0.8658). For F1, 2 versus F3, 4, the AUROC was 0.8238 (0.7529–0.8947). For F1–3 versus F4, the AUROC was 0.8112 (0.7356–0.8868). The Spearman rank correlation coefficient between the APRI and METAVIR F was 0.593 (*P* < 0.001) (Fig 2).

### CPA and SWV for dichotomizing fibrosis stages

The CPA (%, median and range) in each METAVIR F stage were as follows: 3.99 (0.58–10.32) in F1; 9.03 (1.25–25.82) in F2; 24.48 (7.68–56.64) in F3; 29.42 (8.37–64.21) in F4 (overall difference, *P* < 0.001, Kruskal-Wallis test) (Spearman rank correlation coefficient = 0.819, *P* < 0.001). The SWV (m/s, median, and range) in each METAVIR F stage were as follows: 1.21 (1.00–2.43) in F1; 1.54 (0.98–3.58) in F2; 2.37 (0.83–4.05) in F3; 3.04 (1.98–4.56) in F4 (overall difference, *P* < 0.001, Kruskal-Wallis test) (Spearman rank correlation coefficient = 0.734, *P* < 0.001) (Table 1 and Fig 1). The Spearman rank correlation coefficient between the CPA and SWV was 0.706 (*P* < 0.001).

The CPA was used to dichotomize METAVIR F stages with optimal cut-off values of 7.47 for F1 versus F2–4; 12.56 for F1, 2 versus F3, 4; and 15.32 for F1–3 versus F4. SWV was used to dichotomize METAVIR F stages with optimal cut-off values of 1.59 for F1 versus F2–4; 1.73 for F1, 2 versus F3, 4; and 1.96 for F1–3 versus F4. To dichotomize F1 versus F2–4, the AUROC for the CPA was 0.9349 (95% CI: 0.8943–0.9755) and SWV was 0.8434 (0.7762–0.9105) (CPA versus SWV, *P* = 0.0063). For F1, 2 versus F3, 4, the CPA was 0.9436 (0.9091–0.9781), and SWV was 0.8997 (0.8444–0.9551) (*P* = 0.1587). For F1–3 versus F4, the CPA was 0.8647 (0.7944–0.9349) and SWV was 0.9036 (0.8499–0.9573) (*P* = 0.2585) (Table 2 and Fig 2).

**Table 2:** Liver fibrosis dichotomization using the CPA and SWV.

	AUROC^a^	Cut-off	Sensitivity	Specificity	PPV^e^	NPV^d^	DOR^c^
METAVIR F1 vs. F2–4							
CPA^b^	0.9349 (0.8943–0.9755)^g^	7.47	97.1	86.4	70.2	98.9	209.8
SWV^f^	0.8434 (0.7762–0.9105)	1.59	72.8	79.4	91.5	49.1	10.3
METAVIR F1, 2 vs. F3, 4							
CPA	0.9436 (0.9091–0.9781)	12.56	83.5	91.4	93.0	80.3	53.8
SWV	0.8997 (0.8444–0.9551)	1.73	91.4	77.2	74.6	92.4	36.0
METAVIR F1–3 vs. F4							
CPA	0.8647 (0.7944–0.9349)	15.32	69.9	91.7	97.5	39.3	25.6
SWV	0.9036 (0.8499–0.9573)	1.96	100.0	68.1	40.0	100.0	NA^h^

^a^Area under receiver operating characteristic curve;

^b^collagen proportionate area (%);

^c^diagnostic odds ratio;

^d^negative predictive value;

^e^positive predictive value;

^f^shear wave velocity (m/s);

^g^95% confidence intervals are included in the parentheses;

^h^not applicable.

### Statistical analysis

During CPA modeling (R^2^ = 0.609, *P* < 0.001, Table 3), the final multiple regression was not adjusted for SWV because of collinearity with METAVIR F stage and the potential effects on SWV imposed by other factors identified in published reports [7]. The METAVIR F stage and platelet count were the strongest among all other covariates correlated to the CPA through multiple regressions. Through the multiple-regression process, INR and serum albumin and bilirubin levels were excluded because the collinearity with METAVIR F and platelet count weakened their significances.

**Table 3:** Multiple regression analyses for the CPA^a^.

	Univariate		Multiple	
Variable	coefficient	*P* value	coefficient	*P* value
Age (years)	0.367 (0.083)^e^	<0.001		
Male sex (vs. female)	-0.593 (0.104)	0.779		
Body mass index (kg/m^2^)	0.224 (0.307)	0.466		
HCV genotype				
1 (vs. non-1)	-3.025 (2.083)	0.149		
HCV RNA (IU/mL)	0.001 (0.000)	0.947		
METAVIR F^d^ stage (vs. 1)				
2	6.003 (1.830)	0.001	5.163 (1.800)	0.005
3	18.648 (1.953)	<0.001	16.250 (2.104)	<0.001
4	24.696 (2.147)	<0.001	22.225 (2.433)	<0.001
METAVIR A^c^ grade (vs. 0)				
1	7.030 (2.936)	0.018		
≥2	16.706 (3.073)	<0.001		
Steatosis grade (vs. 0)				
1	6.424 (3.318)	0.055		
≥2	9.584 (3.127)	0.003		
INR^b^	55.716 (10.233)	<0.001		
Platelet (10^9^/L)	-0.115 (0.015)	<0.001	-0.037 (0.014)	0.011
White blood cell (10^6^/L)	0.001 (0.001)	0.741		
Hemoglobin (g/dL)	-0.995 (0.635)	0.119		
Sodium (mEq/L)	0.464 (0.453)	0.331		
Albumin (g/dL)	-11.929 (2.718)	<0.001		
Creatinine (μmol/L)	0.062 (0.055)	0.372		
Bilirubin (μmol/L)	0.471 (0.143)	0.001		

^a^collagen proportionate area (%);

^b^international normalized ratio of prothrombin time;

^c^METAVIR A, activity;

^d^METAVIR F, fibrosis;

^e^standard errors of coefficients are included in the parentheses.

Then, the CPA was applied instead of the conventional METAVIR F stage for SWV modeling (R^2^ = 0.492, *P* < 0.001, Table 4). In addition, the CPA was estimated to be strongly correlated to SWV, and necroinflammation measured using METAVIR A grades explained the SWV too.

**Table 4:** Multiple regression analyses for the SWV^d^.

	Univariate		Multiple	
Variable	coefficient	*P* value	coefficient	*P* value
Age (years)	0.022 (0.006)^e^	<0.001		
Male sex (vs. female)	-0.244 (0.138)	0.079		
Body mass index (kg/m^2^)	0.070 (0.019)	<0.001		
HCV genotype 1 (vs. non-1)	-3.025 (2.083)	0.149		
HCV RNA (IU/mL)	0.001 (0.000)	0.246		
CPA^a^	0.045 (0.004)	<0.001	0.036 (0.005)	<0.001
METAVIR A^b^ grade (vs. 0)				
1	0.466 (0.187)	0.014	0.259 (0.160)	0.226
≥2	1.216 (0.194)	<0.001	0.555 (0.187)	0.004
Steatosis grade (vs. 0)				
1	0.350 (0.213)	0.104		
≥2	0.769 (0.201)	<0.001		
International normalized ratio^c^	3.551 (0.683)	<0.001		
Platelet (10^9^/L)	-0.007 (0.001)	<0.001		
White blood cell (10^6^/L)	0.001 (0.001)	0.641		
Hemoglobin (g/dL)	-0.084 (0.042)	0.046		
Sodium (mEq/L)	-0.015 (0.031)	0.633		
Albumin (g/dL)	-0.987 (0.171)	<0.001		
Creatinine (μmol/L)	-0.001 (0.004)	0.902		
Bilirubin (μmol/L)	0.028 (0.010)	0.004		

^a^collagen proportionate area (%);

^b^METAVIR A, activity;

^c^international normalized ratio of prothrombin time;

^d^shear wave velocity (m/s);

^e^standard errors of coefficients are included in the parentheses.

**Fig 3:**
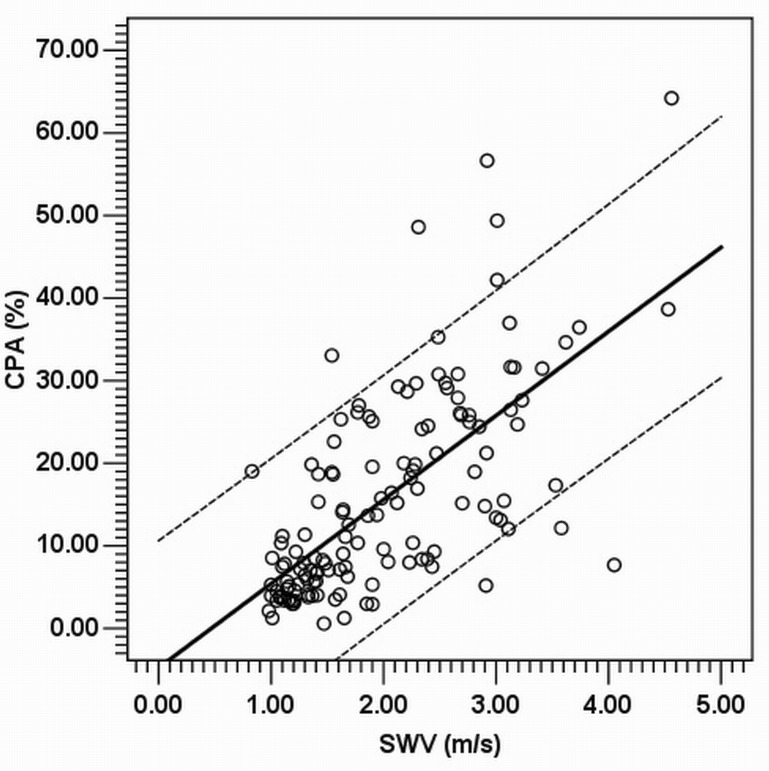
Scatter plot between the CPA^a^ (Y-axis) and SWV^b^ (X-axis). For CPA prediction by using SWV through univariate linear regression, the CPA (%) = -4.768 + SWV (m/s) × 10.184, as a line of best fit (R^2^ = 0.455, *P* < 0.001). Eight of 137 cases (5.8%) exceeded the 95% confidence intervals. ^a^collagen proportionate area; ^b^shear wave velocity.

Finally, to implement the aim for practical settings, the CPA was further modeled from SWV instead of METAVIR F. The CPA could be predicted using SWV alone through univariate linear regression as a line of best fit (R^2^ = 0.455, *P* < 0.001) by using the formula, CPA (%) = -4.768 + SWV (m/s) × 10.184. Eight of 137 cases (5.8%) exceeded the 95% CIs (Fig 3). In addition, through multiple regressions, SWV and platelet count were the strongest again among all other covariates adjusted to model for the CPA. Because platelet count was estimated as the other independent and significant covariate correlated to CPA, the SWV-CPA relationship can be also formulated as CPA (%) = 8.788 + SWV (m/s) × 8.233 + platelet (10^9^/L) × -0.061 (R^2^ = 0.524, *P* < 0.001).

## Discussion

Despite the operator-dependence, the noncategorical CPA applies interactive thresholding to exclude the collagenous structures irrelevant to the disease through digital image analysis, thus providing more refined liver fibrosis evaluation than conventional fibrosis staging. The consistency of the fibrosis ratio is optimal and sensitive particularly for fibrosis surveillance of the liver fibrogenesis bidirectional model. This assists in the monitoring of fibrosis resolution [24] during causality investigation along with relevant molecular pathways and verifying the responses to potential antifibrotic therapy.

Hepatic decompensations and portal-hypertension-related adverse endpoints are fundamental concerns for both clinicians and patients with chronic liver diseases, particularly at the cirrhosis stage. CPA is a promising independent significant discriminator for stratification to identify hepatic decompensation at baseline [25] or over time [10, 26].

The CPA can be used to stratify decompensated cirrhosis into three distinct subgroups in patients with chronic hepatitis B undergoing liver transplantation [25]. The CPA is significantly correlated to liver reserve surrogates, including MELD scores, INR, and bilirubin levels [25].

However, the invasive nature of CPA may compromise the role of the CPA as a universally practical, diagnostic, or prognostic tool in cirrhosis. Therefore, noninvasive measures should be used. Although the CPA is not correlated to necroinflammation [25], which was supported by our study (Table 3), necroinflammation augmented the noninvasive ultrasound-based LS (Table 4).

In addition to the significant correlations at baseline [7], LS dynamics were similar to those of necroinflammatory activities with positive correlations along hepatitis flares [27]. However, necroinflammation is mathematically or statistically unpredictable despite the development of specific cut-off values [28] and a scoring system to predict overrating defined as discordance of liver fibrosis staging (>1 stage) [29].

The observation that the CPA was superior to SWV at fibrosis stages F1 versus F2–4 (Table 2) is supported by reports [7] demonstrating the diagnostic performances (AUROCs) of LS that are unsatisfactory during the dichotomization of low or mild stages or fibrosis strata diagnoses. In addition, the positive necroinflammatory effects on LS tend to be significant only at mild or noncirrhotic fibrosis stages and not in cirrhosis [6]. When dichotomizing F1–3 versus F4, SWV even seemed to have the tendency to be superior to CPA although the *P* value was insignificant (0.2585). Further studies enrolling a larger sample size, especially a larger number of patients with F4 stage, are required to reexamine the potential superiority of SWV over CPA when diagnosing cirrhosis and to perform internal validations on CPA and SWV.

In the multiple-regression model for SWV, the fitness indicated by the R^2^ value (0.492) was moderate. This could be partially explained by the use of categorical METAVIR A grades. Tissue markers that represent real-time hepatic inflammation more strongly than the present case must be developed and applied. Serum ALT levels were not used because the abrupt changes in the levels may not be validly consistent with the real-time hepatic necroinflammation activities. In our observations, ALT levels were nonsignificant when applied instead of METAVIR A to the SWV model.

Our observation that METAVIR F stage and platelet count independently explain the CPA suggests that the CPA can quantify liver fibrosis severity more accurately than METAVIR F staging because CPA considers platelet count. Furthermore, platelet count is correlated to portal hypertension [30]; thus, the CPA is a promising surrogate of the degree of portal hypertension in addition to liver fibrosis. This is also supported by earlier reports that the CPA is a promising predictor of hepatic decompensation and prognosis for stratifying cirrhosis [9–14]. However, for clinical purposes, a linear regression formula to predict the CPA by using noninvasive LSM alone or combined with platelet count is proposed. Although predicting the CPA by combining LS and platelet count (R^2^ = 0.524) is more accurate than by using LS (R^2^ = 0.455), it is acceptable to estimate the CPA from LS alone for liver fibrosis quantification.

In conclusion, this is the first head-to-head comparison of the diagnostic performance in liver fibrosis evaluation between CPA and ARFI elastography in patients with CHC. Both CPA and ARFI elastography are promising diagnostic models feasible for liver fibrosis quantification. During dichotomization, the CPA was superior to ARFI elastography when used to diagnose significant (≥ F2) liver fibrosis. The CPA may be independent of the severe necroinflammation that may augment liver stiffness. CPA values can be derived from LSM. Further development of the liver-stiffness-based predictive model for the CPA is warranted.
